# Effect of MHC and inbreeding on disassortative reproduction: A data revisit, extension and inclusion of fertilization in sand lizards

**DOI:** 10.1002/ece3.9934

**Published:** 2023-03-27

**Authors:** Badreddine Bererhi, Pierre Duchesne, Tonia S. Schwartz, Beata Ujvari, Erik Wapstra, Mats Olsson

**Affiliations:** ^1^ Department of Biological and Environmental Sciences University of Gothenburg Gothenburg Sweden; ^2^ Department of Biology Laval University Quebec Québec Canada; ^3^ Department of Biological Sciences Auburn University Auburn Alabama USA; ^4^ School of Life and Environmental Sciences, Centre for Integrative Ecology Deakin University Waurn Ponds Victoria Australia; ^5^ School of Natural Sciences University of Tasmania Hobart Tasmania Australia; ^6^ School of Biological Sciences University of Wollongong Wollongong New South Wales Australia

**Keywords:** assortative mating, *Lacerta agilis*, major histocompatibility complex (MHC), microsatellite‐screened inbreeding, pre‐copulatory biased paternity

## Abstract

The harmful effects of close inbreeding have been recognized for centuries and, with the rise of Mendelian genetics, was realized to be an effect of homozygosis. This historical background led to great interest in ways to quantify inbreeding, its depression effects on the phenotype and flow‐on effects on mate choice and other aspects of behavioral ecology. The mechanisms and cues used to avoid inbreeding are varied and include major histocompatibility complex (MHC) molecules and the peptides they transport as predictors of the degree of genetic relatedness. Here, we revisit and complement data from a Swedish population of sand lizards (*Lacerta agilis*) showing signs of inbreeding depression to assess the effects of genetic relatedness on pair formation in the wild. Parental pairs were less similar at the MHC than expected under random mating but mated at random with respect to microsatellite relatedness. MHC clustered in groups of RFLP bands but no partner preference was observed with respect to partner MHC cluster genotype. Male MHC band patterns were unrelated to their fertilization success in clutches selected for analysis on the basis of showing mixed paternity. Thus, our data suggest that MHC plays a role in pre‐copulatory, but not post‐copulatory partner association, suggesting that MHC is not the driver of fertilization bias and gamete recognition in sand lizards.

## Graphical abstract

No evidence of inbreeding avoidance in a population of sand lizards (*Lacerta agilis*). In the same population, parental pairs exhibit a disassortative mating pattern with respect to MHC genotypes.

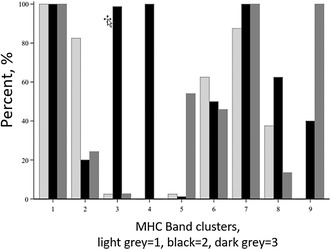

## 1 INTRODUCTION

The effects of close inbreeding have long been recognized, and two main theories have been proposed to explain its negative effects manifested in inbreeding depression and positive effects in situations of heterosis (its converse observed at outbreeding of two inbred F1 lines); (i) at overdominance, there is heterozygote advantage over both homozygotes and (ii) at partial dominance, the heterozygote resembles one homozygote more than the other in some trait of interest. Detrimental fitness effects have been observed at both ends of the inbreeding–outbreeding continuum and have been reported in a wide range of taxa (Amos et al., 2001; Bateson, 1978, 1982; Crnokrak & Roff, 1999; Huisman et al., 2016; Keller et al., 1994; Keller & Waller, 2002; Olsson, Gullberg, & Tegelstrom, 1996; Pusey & Wolf, 1996; Slate et al., 2000). Irrespective of the underlying mechanism, inbreeding can have severe negative effects on fitness, defined as inbreeding depression (Charlesworth & Charlesworth, 1987). At the other genetic extreme, when outbreeding results in detrimental effects on fitness, this has been explained by the breaking up of co‐adapted gene complexes at hybridization between species or diverging conspecific populations (Bateson, 1983). The detrimental effects of outbreeding can then lead to outbreeding depression, although examples are few (Bateson, 1978, 1982; Pusey & Wolf, 1996).

One of the major reasons for studying the inbreeding–outbreeding continuum in evolutionary biology is its potential influence on mating systems and the evolution of inbreeding avoidance mechanisms (Pusey & Wolf, 1996). That said, the assumption that organisms systematically avoid inbreeding ignores the potential costs of outbreeding (Batesson, 1983) and the benefits of inbreeding from inclusive fitness (Bateson, 1983; Lehmann & Perrin, 2003; Kokko & Ots, 2006). In some populations, inbreeding is common both at pre‐copulatory (Duarte et al., 2003) and post‐copulatory stages. The latter occurring through, for example, biasing of sperm competition in favor of less related males (Lane et al., 2007). An important outcome of these studies was to show no clear evidence of inbreeding depression and, hence, no verified selection for inbreeding avoidance, although such effects can go undetected (e.g., Hansson et al., 2007; Keller & Arcese, 1998; Robinson et al., 2012a; Tan et al., 2012). Interestingly, a preference for consanguineous matings can prevail (Bordogna et al., 2016; O'Brien et al., 2019; Robinson et al., 2012b; Schjorring & Jäger, 2007; Thünken et al., 2007, 2011) and be “fine‐tuned” to intermediate levels of inbreeding, referred to as “optimal outbreeding” and ‘optimized’ at first‐second cousin matings (Bateson, 1982). In humans, however, matrimonial practices such as first‐cousin marriages in Pakistani and Moroccan culture result in severe loss of fertility and increased risk of malformations (Cheffi et al., 2022; Iqbal et al., 2022).

Inbreeding avoidance may occur through a variety of mechanisms (Pusey & Wolf, 1996), including dispersal (Bollinger et al., 1993; Greenwood, 1980; Pusey, 1987), kin recognition (Gerlach & Lysiak, 2006; Hoffman et al., 2007; Sherborne et al., 2007), and polyandry (Bichet et al., 2014; Bretman et al., 2004; Firman & Simmons, 2008; Foerster et al., 2003; Olsson, Shine, et al., 1996; Simmons et al., 2006; Tregenza & Wedell, 2002). One proposed predictor of genetic relatedness for mate discrimination is similarity at the major histocompatibility complex (MHC) (Brown & Eklund, 1994; Penn & Potts, 1999; Potts & Wakeland, 1993). Furthermore, mate choice for complementarity of MHC genes to gain immunological fitness benefits for the offspring have long been recognized. MHC codes for highly polymorphic glycoproteins that are expressed on cell surfaces. These molecules are involved in initiating immune responses by binding pathogen‐derived peptide fragments (Janeway Jr. et al., 2001). Parental pairs that are relatively dissimilar at the MHC are expected to have relatively more MHC‐heterozygous offspring with the potential to resist a wider range of pathogens and parasites. Alternatively, MHC‐based disassortative mating may be advantageous by producing offspring that can resist evolving parasites through rare allele advantages, the red queen hypothesis. (i.e., an arms race of host–parasite interactions; Penn & Potts, 1999). So, even if disassortative mating patterns are found with respect to genetic relatedness overall, they may represent artifacts of a general preference for MHC dissimilarity. It is challenging to identify the primary selective pressures underlying non‐random associations with respect to genetic similarity, at either the MHC or at the genome‐wide level. The evolutionary significance of MHC‐based mate choice may, therefore, be disentangled from that based on genome‐wide similarity by contrasting observations of mating patterns based on measures of MHC‐similarity vs, a ‘neutral’ genomic marker, such as microsatellites. That is the aim of this study.

## 2 MATERIALS AND METHODS

### 2.1 The sand lizard (*Lacerta agilis*) model system

Sand lizards are small ground dwellers (up to 20 g) and have one of the largest distributions of any reptile species, ca. 8000 × 5000 km (Bischoff, 1984), with their main distribution in central Europe. In Sweden, the distribution is fragmented, and the genetic variation is low compared to continental Europe (Gullberg et al., 1999). In our study population (Asketunnan; N570 22′ E110 58′), matings between close kin are detrimental as revealed by malformations in inbred offspring (Bererhi et al., 2019; Olsson, Gullberg, & Tegelstrom, 1996). Females lay a single annual clutch of five to 15 eggs, depending on female body size (Olsson & Shine, 1997). Approximately 1 week before egg laying, when females show egg contours along their abdomens, gravid females were captured by noose or hand and brought to facilities at the University of Gothenburg, Sweden. They were marked by claw/toe‐clipping for identification (Olsson, 1994) and kept individually in cages (40 × 50 × 60 cm) containing a flat rock, placed on wet sand for egg laying, and a 40‐W spotlight at one end to allow thermoregulation. Eggs were collected within hours of laying and incubated at 25°C, which optimizes hatching success and minimizes developmental asymmetries in this species (Zakharov, 1989). All clutches were incubated individually in separate boxes, all in the same incubator. Eggs hatched after approximately 40 days. The juveniles were measured snout to vent to the nearest mm, weighed to the nearest 0.001 g and marked by claw/toe‐clipping. Hatchlings were then blood (or tail tip, tails are regenerated in Lacertid lizards) sampled prior to release at random sites at the study site, whereas unhatched embryos were kept for DNA sampling.

Females have significantly smaller home ranges than males, ca. 160 m^2^ and 1100 m^2^, respectively, on average (Olsson, 1994). Females are visited by courting males (Olsson, 1994), and females do not appear to exert mate choice with respect to exclusively male traits, such as nuptial coloration (Olsson, 1994; Olsson, Gullberg, et al., 1994; Olsson, Norberg, et al., 1994). Furthermore, female sand lizards do not appear to reject close relatives as mates (Olsson, Gullberg, & Tegelstrom, 1996). A female's offspring viability is positively correlated with her number of mating partners, suggesting the existence of post‐copulatory mechanisms of mate discrimination. These would be potentially based on genetic compatibility (Olsson & Madsen, 2001), perhaps also at the MHC. Indeed, genetic similarity between copulating partners, calculated using DNA fingerprinting and microsatellites, was negatively correlated with the proportion of sired offspring in multiply sired clutches (Olsson, 1994; Olsson, Gullberg, et al., 1994; Olsson, Gullberg, & Tegelstrom, 1996; Olsson, Norberg, et al., 1994; Olsson & Shine, 1997; Olsson, Shine, et al., 1996).

We were especially keen to understand whether MHC contributes more, or more important, genetic information than mere relatedness at ‘neutral’ loci. We, therefore, contrasted the degree of MHC relatedness of parental pairs against expectations under random mating. To do this, we used microsatellite and MHC‐derived similarity indices, obtained over a four‐year period for 153 mated pairs using a single clutch per pair. In addition, we explored MHC genotype profiles in the population, to assess the potential existence of distinct clusters of parental genotypes and determine if parental combinations occur at random with respect to these loci combinations.

The occurrence of inbreeding in our sand lizard population has been assessed before through heterozygosity‑heterozygosity correlations at microsatellite loci and the calculation of the g2 parameter, as an estimate of identity disequilibrium across the genome. The results of both approaches were positive and significant, indicating a signature of inbreeding (Bererhi et al., 2019).

#### 2.1.1 Molecular genetics protocols

For a detailed description of DNA extraction, genotyping and paternity assignment, see Olsson et al. (2011). In brief, DNA was extracted from blood and tissue samples, and up to 21 microsatellite loci (minimum 17) were used for paternity assignment (Table S1). In total, DNA was obtained from 4534 individuals, including juveniles, adults, and unhatched embryos. Of 452 non‐hatched eggs, 204 were successfully genotyped. The remaining 248 embryos could not be genotyped due to low‐quality DNA. In total, 2757 juveniles of 3627 hatched eggs were paternity‐assigned (Olsson et al., 2011). The adults from 1998 were first used to test for Hardy Weinberg equilibrium in GENEPOP (version 4.2) with Markov chain parameters set to 1000 dememorization steps, 200 batches, and 1000 iterations (Raymond & Rousset, 1995; Rousset, 2008), with *p*‐values corrected for multiple tests using False Discovery Rates (Benjamini & Hochberg, 1995). No *p*‐values were lower than the critical FDR value after corrections. Thus, the considered loci were in Hardy Weinberg equilibrium (Bererhi et al., 2019).

#### 2.1.2 Microsatellite relatedness (r) calculation

Over 750 adults captured over a ten‐year period were genotyped using the above‐mentioned microsatellite methods. Relatedness was estimated using the relatedness coefficient ‘r’ (Wang et al., 2011) for all hypothetical pairs and pairs that successfully produced eggs (299 females and 454 males). The relatedness coefficient was calculated in SPAGEDI version 1.5 (Hardy & Vekemans, 2002). This ‘r’ coefficient estimates the proportion of shared alleles for a pair of individuals, while considering the whole sample frequencies for the same alleles. This results in possible ranges between −1 and 1. These pairs were also genotyped for MHC loci, to ensure that the same pairs are used for both MHC and microsatellite analyses. In total, 79 males and 69 females were included in the randomization procedure, 153 unique pairs, each used once only.

#### 2.1.3 MHC band sharing using RFLP

Restriction fragment length polymorphism (RFLP) analysis was developed in the 1980s using restriction endonucleases which cleave DNA molecules at specific sites and species‐specific cloned DNA probes are then used to detect specific homologous DNA fragments. RFLPs have genetic characteristics that made them attractive genetic tools, such as lack of dominance, multiple allelic forms, and absence of pleiotropic effects. In our study, species‐specific RFLP MHC probes were developed in 1998–99, (see Madsen et al., 2000, for a detailed description of methods used). We are of course aware that since then, RFLP has become largely obsolete due to the emergence of next‐generation DNA sequencing. In our case, however, we find further analyses of our RFLP data motivated for the following reasons: (i) In Olsson et al. (2005), we describe a genetic fragment in our analyses (the ‘O‐genotype’), whose carriers had less ectoparasites, were more colorful, more successful at mate acquisition and mate guarding and, ultimately, had higher genetic reproductive success (molecularly assigned; Olsson et al., 2005). (ii) In the laboratory, females were more attracted to odor samples obtained from males with less similar MHC RFLP bands to their own, relative to males with more similar bands (Olsson et al., 2003). (iii) With a much smaller sample, we showed that pair formation with regard to MHC similarity can be non‐random in this study population (Olsson et al., 2003). Thus, these results seem to strongly indicate that our RFLP MHC genotypes capture significant immunogenetic biology.

RFLP of sand lizard MHC class 1 genes was analyzed using species‐specific probes for 290 adults sampled in 1998–2000. MHC band sharing was calculated for the 153 unique pairs of mated individuals, 79 males and 69 females, using the band sharing index D = 2 F_ab_/(F_a_ + F_b_) (Wetton et al., 1987). Here, F_a_ and F_b_ represent the number of bands in individuals a and b, and F_ab_ the number of shared bands. In total, 9 bands were used for the band sharing analyses.

#### 2.1.4 Randomization procedures and association between relatedness and MHC band sharing

- Data description

To identify disassortative mating patterns, two sets of simulations were run, one for MHC band sharing (D) and one for microsatellite relatedness (r). Mating data were used for 4 years from 1998 to 2001 and included 148 adults (79 males and 69 females). These 5 years represent the period for which individuals were genotyped for both microsatellite and MHC genomic regions. The data for each year were analyzed separately to ensure that randomized pairs were also ‘real life’ potential pairs. Each mated pair only appeared once across years in the data set to ensure observation independence.

b Simulation procedure

Each simulation involved the randomization of mated pairs and was performed in three steps:

1. The sum of D's for (MHC), or r's (for microsatellites), using all observed parents was calculated for each year.
2. Male and female genotypes were paired randomly for each year, and the sum of D's, or r's, using all random pairs was calculated.
3. The sums for the observed and randomized pairs were compared.

A total of 10,000 simulations were run for each year. For each set of 10,000 simulations, the number of times that the observed pairs had a similarity sum equal to or smaller than that of randomized pairs was calculated and that number was then divided by 10,000, yielding a *p*‐value. Thus, the *p*‐value represents the percentage of times that the observed sum of D's, or r's, was equal to or smaller than the sum of D's, or r's, of the randomized pairs. Importantly, note that in each randomization, the total number of pairs and the number of pairs associated with each male and each female are identical to those of the empirical pairings. All calculations were performed in Maple 13 programming language. Once a *p*‐value was obtained for each year, a compound *p*‐value was calculated for all years using two approaches: Fisher's combined probability test (Mosteller & Fisher, 1948), and Lancaster's statistic T (Chen, 2011; Zaykin, 2011). The reason for using both was that Chen found that Lancaster's generalization of Fisher's test was more powerful than some other tests in common use for combining *p*‐values (e.g., the weighted *Z*‐test, Zaykin, 2011). We followed Chen (2011) by transforming *p*‐values to chi‐square variables by an inverse chi‐square transformation with the degrees of freedom equal to the sample size each year of the study, i.e., Lancaster's statistic *T* = Σ [*X* ^2^_*(ni)*_]^−1^ (1 – *p*_i_) with the distribution *T* ~ *X* ^2^_(Σ*n*i)_.

Finally, a Spearman's rank order correlation analysis was performed between r‐ and D‐values, using 180 unique parental pairs. These were obtained from the data collected between 1998 and 2003. A non‐parametric correlation was used due to non‐normal data distribution.

#### 2.1.5 MHC profiles

The population level MHC profiles were explored in the clustering program FLOCK 3.1 (Duchesne & Turgeon, 2012). We specifically analyzed the presence of distinct groups of MHC genotypes in the population based on the presence or absence of RFLP bands and if mating is random among these potential groups. This was achieved by comparing the frequencies of the observed parental combinations among clusters with those expected under random mating using a *χ* ^2^ test.

#### 2.1.6 Statistical methods

- Choice of analytical tools

All statistical analyses were performed in SAS (Statistical Analysis System) 9.4. Parental pair was first analyzed as a categorical variable (empty model), followed by entering year, juvenile sex, and r (all indicated as potential predictors in previous work). Model fit was compared using Log Likelihood ratio tests (LL ratio test). A LL ratio represents the difference between the −2 Log Likelihoods of two models (Wang et al., 2011). In addition, model fit was compared using the information criterion Akaike information criterion (AIC). The aim with this approach is to differentiate between MHC compatibility and straight inbreeding effects of microsatellite relatedness, while accounting for environmental heterogeneity (year) and potential sex‐specific effects.

b Using mixed paternity clutches to analyze fertilization bias: pros and cons

In our data set, it is not possible to determine whether single‐paternity clutches are the result of a single copulation opportunity for a female, are due to pre‐copulatory multi‐male processes (e.g., potentially strong female choice or male–male competition), or whether cryptic female choice or sperm competition result in complete paternity bias from MHC complementarity or inbreeding avoidance at large. Thus, such clutches, although the most informative under completely controlled laboratory matings, will potentially bias an interpretation of paternity data with respect to MHC or genomic relatedness at large in the wild. The strictest approach based on confirmed paternity would, therefore, be to focus entirely on multiple‐paternity clutches, which confirms that a female has been inseminated by the identified fathers in the clutch but leaves open the opportunity to analyze paternity bias relative to other males' and female's genotypes. Therefore, we specifically analyzed patterns of parentage in mixed‐paternity clutches to achieve highest level of relatedness effects at the MHC, and of r, on relative fertilization success of competing males, and their relatedness to the female (as per Olsson et al., 1997).

We assessed the effect of MHC band similarity of mates on fertilization success by selecting 56 mixed‐paternity clutches for which we had MHC band data assigned for all males. Each clutch was represented by one female in a given year and randomly selected males for a total of 40 females and 39 males. For each clutch, mean D was calculated, including all values between the mother and each father that contributed to the clutch. This clutch average was then used to calculate a relative D for each male, as individual D minus average D. Thus, positive values would indicate more MHC similarity between a father and a mother relative to other fathers in that clutch. The relative proportion of sired offspring for every male in every clutch was calculated by subtracting the expected proportion of sired offspring in a clutch from the observed proportion. The expected proportions of sired offspring were calculated based on equal parentage probability for all males. This method (Lane et al., 2007; Olsson, Shine, et al., 1996) allowed us to estimate the effect of pairwise MHC band sharing effects on fertilization success relative other males.

General linear mixed‐effects models were used to test the effect of relative MHC D (fixed effect) on the relative proportion of sired offspring. Relative MHC D was added as a fixed effect to a model that included both random effects (intercept only model, maternal, and paternal IDs) with model fits compared using LL ratio tests and AIC comparisons.

## 3 RESULTS

### 3.1 Mating patterns and relatedness

**FIGURE 1:**
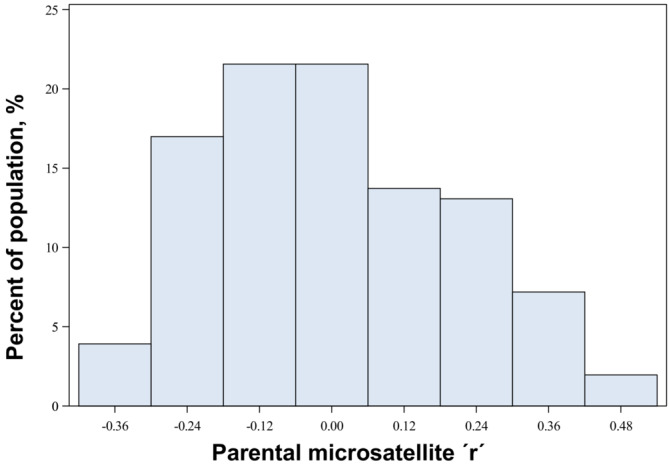
Distribution of the parental relatedness coefficient (r) in the study population, calculated using microsatellite data (*n* = 153; Mean = −0.00082; *SD* = 0.2007; Maximum value = 0.4941; minimum value = −0.4129).

**FIGURE 2:**
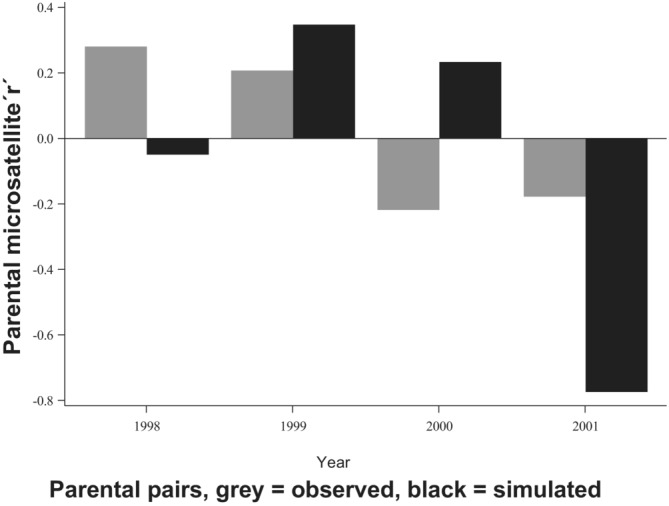
Comparison between the observed and the simulated parental relatedness in the study population for 4 separate years. The observed relatedness was estimated as the sum of r's of the parental pairs during each year. The simulated r represents the average sum of r's after 10,000 simulations of random pairing. Note that 163 pairings are included in the chart, instead of 153 used in the analyses. This difference is due to the fact that 10 pairs were removed for statistical analyses, as they appeared in more than 1 year.

The mean microsatellite relatedness (r) for the 153 parental pairs included in the analyses was – 0.00082 (*n* = 153; SD = 0.20073; Range = 0.907; Min = − 0.412; Max = 0.494) (Figure 1). No indication of disassortative mating was found according to microsatellite r (*χ* ^2^_8_ = 5.08; *p* = .75), i.e., the relatedness of the empirical pairs was not significantly different from expectations under random mating (Table 1; Figure 2).

**TABLE 1:** Results after 10,000 pairing simulations using MHC and microsatellite data.

		MHC		Microsatellites	
Year	Pairs	Sum D	*p*‐value	Sum r	*p*‐value
1998	29	18.4691	.4171	0.1239	.4579
1999	32	21.801	.6607	0.1323	.604
2000	41	25.3537	.0115	0.0869	.5267
2001	51	31.6427	.0515	−0.6335	.5413

*Note*: The table shows the number of pairs used for each year. The sums of D's, and r's, for the empirical pairs are listed for every year, with the corresponding *p*‐value. Combined *p*‐values are given in the Results section.

**FIGURE 3:**
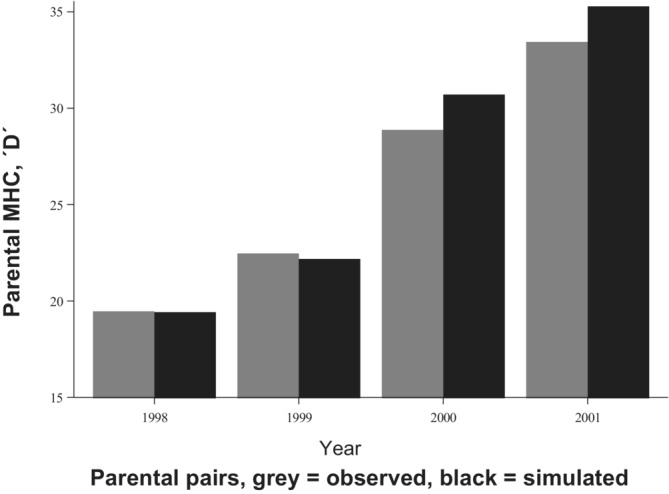
Comparison between the observed and the simulated parental MHC band sharing (D) in the study population during 4 years. The observed D was estimated as the sum of D's of the parental pairs during each year. The simulated D represents the average sum of D's after 10,000 simulations of random pairing. Note that 163 pairings are included in the chart, instead of 153 used in the analyses. This difference is due to the fact that 10 pairs were removed for statistical analyses, as they appeared in more than 1 year.

Empirical pairs had lower MHC band similarity than randomized pairs (*χ* ^2^_8_ = 17.44; *p* = .026; Table 1, Figure 3) using Fisher's test for combined *p*‐values across all 4 years. However, using Lancasters' T, the overall combined *P*‐value across all 4 years fell short of significance (*p* = .061). Furthermore, on closer scrutiny, in the first 2 years (1998–1999), MHC similarity between empirical and simulated pairs was the same or slightly (non‐significantly) biased towards greater MHC similarity between empirical pairs. In contrast, in year 2000–2001, the pattern was consistent with lower MHC bias in empirical than in simulated pairs, and in those 2 years, Lancaster's *T* (i.e., with *p*‐values combined) was statistically significant (*p* = .029). Previous results show similar year‐to‐year mating system effects (higher degree of polyandry) in years with more benign climate for ectotherm activities, which we will return to in the Discussion (Olsson et al., 2011).

**FIGURE 4:**
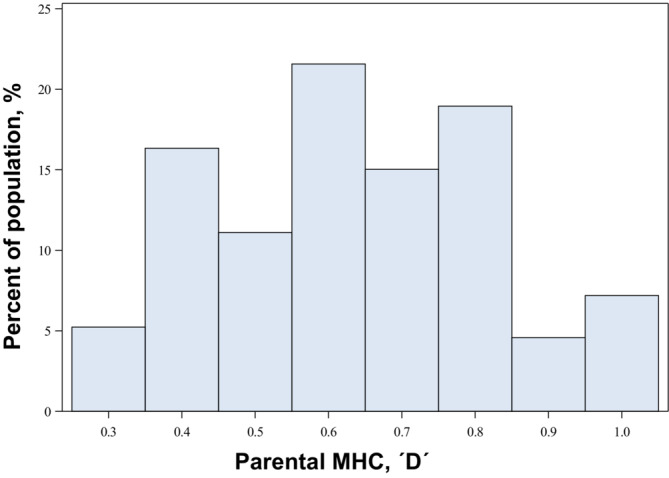
Distribution of the parental MHC band sharing index (D) for the 153 pairs included in the analyses (*n* = 153; Mean = 0.6357; *SD* = 0.1929; Maximum value = 1; minimum value = 0.25).

**FIGURE 5:**
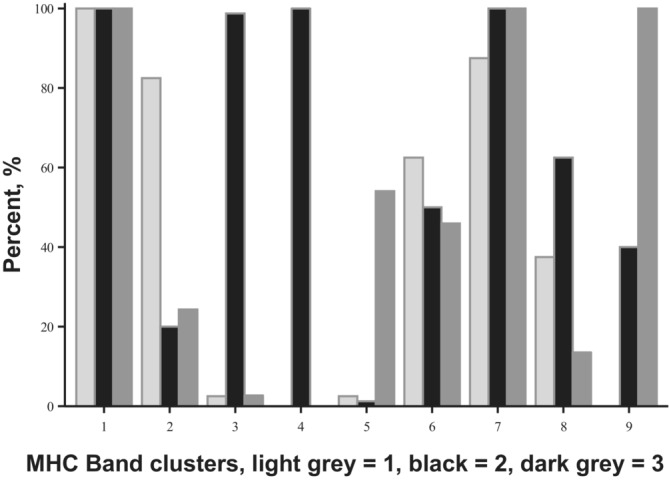
The three MHC genotype clusters are shown in different colors. The X‐axis represents the nine MHC bands used. The Y‐axis shows the percentage of the individuals with each band in each cluster.

The effect of relative MHC D on the relative proportion of sired offspring was non‐significant (*n* = 56; estimate: 7.13, 95% confidence limits ‐ 38.01–52.27; LL ratio test: *χ* ^2^1 = 0.1, *p* = .751; Δ AIC = −1.9). Mean MHC band sharing (D) was 0.635 (*n* = 153; SD = 0.192; Range = 0.750; Min = 0.25; Max = 1) (Figure 4). The FLOCK results indicate that the MHC genotypes were grouped in three clusters with different band compositions (Figure 5). Of the 9 MHC bands used, one band (band 1) is found in all individuals of the three clusters and, thus, does not provide any information. Similarly, band 7 provides very little information, as it is found in almost all individuals. Band 4 is only found in one cluster of genotypes, and band 3 is almost exclusively found in cluster 2. The latter two bands also appear to be tightly correlated. In other words, these bands do not only appear almost exclusively in cluster 2, but they are also found in all individuals within the cluster (Figure 5). Finally, the parental pairs among the three clusters did not mate disassortatively (χ^2^_4_ = 5.69; *p* = .2232).

**FIGURE 6:**
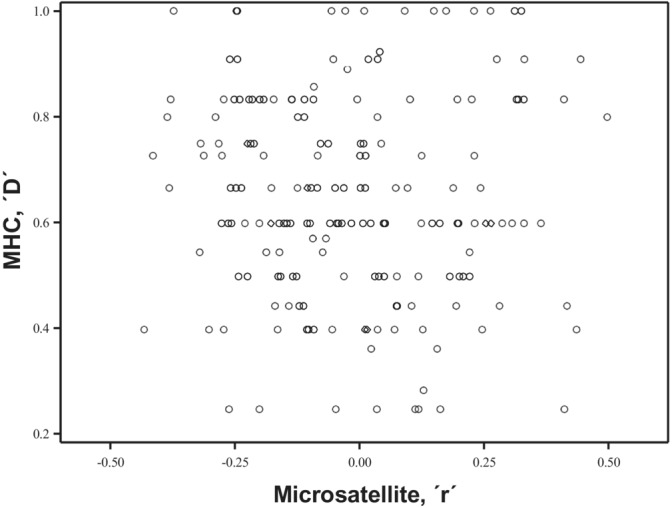
Scatter plot of the relationship between MHC band sharing (D) and relatedness (r) for 180 unique pairs, between 1998 and 2003 (*n* = 180; *r*_*s*_ = −0.065; *p* = .3835).

There was no significant correlation between MHC band sharing (D) and microsatellite relatedness (r) (*n* = 180; *r*_*s*_ = −0.065; *p* = .3835) (Figure 6).

## 4 DISCUSSION

Our primary objective was to investigate the mating patterns in the sand lizard population of Asketunnan, Sweden, with respect to overall inbreeding avoidance (i.e., with respect to ‘r’) and the role of MHC similarity (D) as a cue for mate choice relatedness. To avoid bias in single‐paternity clutches from incomplete copulation history of females, we used clutches with multiple paternity to assess bias in fertilization with respect to ‘genome‐wide’ relatedness (using microsatellite, r) and with respect to MHC similarity of partners and competitors. We found no indication of disassortative mating according to relatedness, using the coefficient of microsatellite relatedness ‘r’ at 21 microsatellite loci to represent the background level of relatedness across the genome. This result suggests that consanguineous matings are not avoided in the population and is consistent with a number of studies in which no or weak evidence of inbreeding avoidance has been found (Duarte et al., 2003; Hansson et al., 2007; Keller & Arcese, 1998; Lane et al., 2007; Mainguy et al., 2009; Robinson et al., 2012a; Tan et al., 2012).

In a previous study on the same population, we showed that offspring standardized heterozygosity SH (Coltman et al., 1999) was positively associated with hatching success (Bererhi et al., 2019). That said, it is important to distinguish between selection on “being inbred” and selection on “inbreeding” (Troianou et al., 2018). Pedigree‐based inbreeding coefficients are approximations of the proportion of the genome that is autozygous (Franklin, 1977; Hill & Weir, 2011). Therefore, identity by descent is expected to vary for offspring in the same pedigree. For example, computer simulations show that marker‐based heterozygosity metrics better predict the realized proportion of the genome that is identical by descent than pedigree‐based inbreeding coefficients (Kardos et al., 2015). Furthermore, Hemmings et al. (2012) demonstrated empirically that marker‐based approaches can be more informative than pedigree‐based methods by estimating realized homozygosity instead of expected genome‐wide homozygosity. Even though we used a molecular marker‐based estimate of relatedness, instead of a pedigree approach, the heterozygosity levels of two siblings with known parental relatedness may still differ substantially. Thus, estimating realized homozygosity may serve as a means of measuring the effects of being inbred, whereas expected homozygosity relates to parental relatedness and its expected effects on offspring viability. Perhaps, more importantly, distinguishing between selection on being inbred and selection on inbreeding may help with addressing cases in which there is no evidence of inbreeding avoidance in spite of strong detrimental effects of being inbred (Troianou et al., 2018).

Individuals tended to have mating partners with whom they shared less MHC bands than expected under random mating, but there was no significant correlation between microsatellite relatedness and the number of shared MHC bands. Thus, based on our findings, we can exclude MHC genotypes as a means by which – strictly – inbreeding can be avoided in the population. Similar divergent patterns between relatedness at the MHC and overall genomic similarity have been reported elsewhere (Juola & Dearborn, 2012; Laundry et al., 2001; Sepil et al., 2015; Sin et al., 2015). Notably, Sin et al. (2015) found divergent mating patterns using MHC class II similarity and microsatellite‐based genetic similarity in a wild population of European badgers (*Meles meles*). In this population, parental pairs exhibited assortative mating at MHC genes and disassortative mating at microsatellite‐based genetic similarity. Therefore, even if overall genetic relatedness can be mediated by MHC genotypes (Penn & Potts, 1999), they do not appear to be an exclusive means of inbreeding avoidance, highlighting the importance of considering the existence of other important, potentially species‐specific, polymorphic signals used for inbreeding avoidance. Other work more targeted towards sexual selection on MHC have shown significant pre‐copulatory sexual selection against certain male haplotypes in Soay sheep (*Ovis aries*), and post‐copulatory disassortative sexual selection and strong inbreeding avoidance (Huang et al., 2021). In gray mouse lemurs (*Microcebus murinus*), researchers failed to find pre‐copulatory MHC mate choice but identified a paternity advantage of fathers with more MHC ‘supertypes’ (Schwensow et al., 2008). Furthermore, in the Tasmanian devil (*Sarcophilus harrisii*), Russell et al. (2018) demonstrated disruptive assortative mating for MHC genotypes such that when a female had fewer MHC types her partners had relatively more, and vice versa. House mice (*Mus musculus domesticus*), a species that appears to use MHC‐related cues for inbreeding avoidance (Potts et al., 1994; Potts & Wakeland, 1993), was shown to use the major urinary protein (MUP) gene cluster for kin discrimination when genome‐wide similarity was controlled for (Sherborne et al., 2007). Thus, the complexity of multiple odor molecules also makes it harder to differentiate between straight‐forward inbreeding avoidance and choice, for e.g., MHC complementarity.

Females prefer MHC dissimilar males in staged mating trials (Olsson et al., 2003), but this may not necessarily imply that they exert mate choice with respect to MHC genotypes in the wild, as this could be exerted by males; male body mass was negatively correlated with partner band sharing, suggesting that dominant, larger males may choose, or spend more time with, more MHC‐dissimilar females (Olsson et al., 2003). That said, further support on the importance of our previous work on the role of female choice on MHC odors in male femoral pore secretions come from proteomics research by Ibánez et al. (2022). The Ibánez work shows that femoral gland secretions in *Lacerta agilis* have proteins that are not used in chemical communication but are consistently related to the immune system, in particular the MHC. The authors describe a similar observation from work on Galapagos marine Iguanas. Thus, this supports our observation of MHC variability being involved in mate discrimination in this species. However, our field data seem to suggest that pair‐related MHC bias may not always be a consistent pattern among years, which agrees with our previous work showing that several aspects of mating system biology, such as degree of polyandry, vary between years depending on climate. In warmer years, when ectotherms are more active, the level of polyandry and the degree to which multiple males sire offspring within the same clutch increases (Olsson et al., 2011), and perhaps, similar effects can be seen with respect to selecting partners based on MHC profile.

In our study, gravid females were brought to the animal facility from the wild prior to egg laying and thus identification of mated pairs prior to fertilization is dependent on field‐based observations (but see Olsson et al., 2019). Consequently, mate choice for dissimilar MHC bands may still have taken place after copulation through cryptic female choice. This may have occurred if the most MHC‐similar males had failed to fertilize at least one egg, rendering them invisible in our analyses. Controlled mate choice experiments would be valuable for further determining the stage of the reproductive process at which disassortative mating/fertilization occurs. Second, the randomization procedure only included the individuals that were captured and genotyped at both the microsatellite and MHC regions, excluding some potential mates each year. Nonetheless, based on our previous observations of pair associations (Olsson et al., 2003), combined with our randomization procedure that included another 3 years of mating data, we are confident that mating pairs in the study population are less MHC‐similar than average. Third, our band sharing analysis was performed using RFLP. This limits our understanding of the functionality or selective advantages underlying the observed disassortative mating pattern. The use of peptide‐binding region sequences and amino acid compositions is likely to improve measures of the degree of functional differences between mated pairs that share different sets of RFLP bands. The use of amino acid composition would allow us to differentiate between mate choice based on maximal dissimilarity and mate choice based on optimal dissimilarity at peptide‐binding regions, thus providing a promising basis to advance our understanding of immunocompetence‐based mate choice in reptiles. Evidence of disassortative mating according to MHC similarity was found in both Atlantic salmon (*Salmo salar*) (Laundry et al., 2001) and tuatara (*Sphenodon punctatus*) (Miller et al., 2009) only when differences in amino acids were considered, instead of differences in the number of shared alleles. Regardless of the potential functional differences between the nine bands that we used, the fact that we found no evidence of inbreeding avoidance using microsatellites lends strong support to the idea that the disassortative MHC‐based mating pattern is linked to immunocompetence directly, instead of representing a general mechanism of inbreeding avoidance.

The MHC profile analyses showed the existence of three clusters of MHC genotypes, with no evidence of either assortative or disassortative mating among them. Bands 1 and 7 convey no or little information, as they are present in almost all genotyped individuals. However, bands 3 and 4 are strongly correlated and found almost exclusively in cluster 2, exclusively for band 4. These three clusters of genotypes may be maintained through natural selection, with certain band profiles conferring higher fitness than others. Alternatively, the clustering may be due to linkage disequilibrium.

The discovery of MHC expression on gamete surfaces (e.g., Mori et al., 1990; review in Kekäläinen & Evans, 2018) led to predictions and expectations to find MHC influencing the fertilization process and deviations from random fertilization success with respect to MHC (Kekäläinen & Evans, 2018). These predictions have met with mixed results. Wedekind et al. (2004) found no effects of MHC on cryptic female choice processes in whitefish (*Coregonus* sp.). Since then, several research groups have established HLA‐dependent cervical mucus‐effects on sperm function with the assumption that this will increase reproductive success in males with boosted sperm performance. Gamete‐level immunogenetic incompatibility have also been found on risk of infertility in humans (Jokiniemi, Kuusipalo, et al., 2020; Jokiniemi, Magris, et al., 2020), and cryptic haplo‐specific gamete selection have been described resulting in offspring with optimal MHC immune genes (Lenz et al., 2018). Thus, there is evidence both for and against MHC molecules as a general cue to fertilization success. We found no evidence of biased paternity according to MHC similarity in mixed‐paternity clutches. Based on this finding, the disassortative mating pattern that we found likely occurs at the pre‐copulatory level, confirming the observations of non‐random pair formations according to band similarity and the female preference for odor samples obtained from more MHC‐dissimilar males relative to males with more female‐similar bands (Olsson et al., 2003).

In summary, we found no evidence of disassortative mating according to microsatellite relatedness. MHC‐based mate choice appears to be independent from choice based on overall genetic similarity, suggesting caution when inferring genetic similarity using genomic regions that are under different levels of selective pressures. We provide evidence that MHC‐based mate choice occurs at the pre‐ but not post‐copulatory level in this study population.

## AUTHOR CONTRIBUTIONS

**Badreddine Bererhi:** Conceptualization (equal); data curation (equal); formal analysis (lead); investigation (equal); methodology (lead); project administration (equal); software (equal); validation (equal); visualization (lead); writing – original draft (lead); writing – review and editing (lead). **Pierre Duchesne:** Formal analysis (equal); methodology (equal); software (equal); visualization (equal). **Tonia Schwartz:** Data curation (equal); formal analysis (equal); methodology (equal); software (equal); validation (equal); visualization (equal); writing – review and editing (supporting). **Beata Ujvari:** Data curation (equal); formal analysis (equal); methodology (equal); software (equal); visualization (equal). **Erik Wapstra:** Data curation (equal); funding acquisition (equal); investigation (equal); project administration (equal); resources (equal); supervision (equal); validation (equal); visualization (equal); writing – review and editing (supporting). **Mats Olsson:** Conceptualization (equal); data curation (equal); funding acquisition (equal); investigation (equal); methodology (equal); project administration (equal); resources (equal); supervision (lead); validation (equal); visualization (equal); writing – review and editing (supporting).

## CONFLICT OF INTEREST STATEMENT

The authors declare no conflicts of interest.

## Supporting information


Table S1

## Data Availability

Data will be made available on Dryad digital repository. The data used in this study are available on Dryad. DOI: https://doi.org/10.5061/dryad.6djh9w15d.
