# Supplementary material for: Effect of MHC and inbreeding on disassortative reproduction: A data revisit, extension and inclusion of fertilization in sand lizards
Source: Ecol Evol. 2023 Mar 27;13(3):e9934. doi: 10.1002/ece3.9934 (PMC10041550; doi:10.1002/ece3.9934)
Supplement: Supplementary file 1 — Table S1 [file ECE3-13-e9934-s001.pdf]

EVOLUTION
INTERNATIONAL JOURNAL OF ORGANIC EVOLUTION

SSE
SOCIETY for the STUDY of EVOLUTION

Free Access

IN HOT PURSUIT: FLUCTUATING MATING SYSTEM AND SEXUAL SELECTION IN SAND LIZARDS

Mats Olsson, Erik Wapstra, Tonia Schwartz, Thomas Madsen, Beata Ujvari, Tobias Uller

First published: 05 October 2010 | https://doi.org/10.1111/j.1558-5646.2010.01152.x | Citations: 44

Table 1. Details of microsatellite loci used for parentage analysis. Size range of alleles (Size), number of alleles (*N*), polymorphic information content (PIC), and the observed heterozygosity H_O, based on all the entire dataset of 3967 genotyped individuals.

Locus	Fluorophore-primer sequence 5′–3′	PCR[6]	Size	*N*	PIC	H_O
LA58[1]	FAM-CAGTTCTGGGGATTTTCTCCTAC<br>CATTGTAATTGGAGCACAAAGC	Mplx2	165–179	6	0.575	0.645
LA64[1]	PET-AGATGCTGAACTACCAGCTTGC<br>GCTATCCTGGCTGACCATTAAG	Mplx3	185–192	3	0.277	0.348
LA1-KB	FAM-AGGTTTCCTGGCTTGGAG<br>ATTTGCACAAAACAGCAGC	Mplx3	101–135	15	0.821	0.843
LA2-AG[2,5]	GCTTAAATTGGAACCAGATTG<br>FAM-AAGCAGCCAGAACACAGAG	Mplx5	187–193	4	0.351	0.39
LA3-AG[2]	AGTAGGAGCGAGAAGAATCAG<br>NED-GACATATGGCAGAAAGAGCAG	Mplx4	158–188	8	0.739	0.786
LV4-72[3]	NED-CAAAGCCAAAGAAGGCTCTC<br>CTTTGCAGGTAACAGAGTAGTTC	Mplx4	97–113	5	0.668	0.717
LV-4-X[3]	PET-TGAAACATGGATTAGAGGCTGA<br>ACTCCTTGCGTGGCATAAAA	Mplx4	165–172	6	0.323	0.352
Lvir7[4,5]	VIC-TCGACAGCTTGCAGGCTTGAC<br>GAAGGGCTCTTCCAGACACTG	Mplx5	320–360	8	0.732	0.779
Lvir17[4,5]	FAM-AGCTCTGGATCGAGACAACCTGG<br>TCTCTGAAGGAGACCGGCTCC	Mplx5	312–334	5	0.628	0.666
